# It Takes Two to Tango: A New Partner in Amylose Synthesis

**DOI:** 10.1371/journal.pbio.1002079

**Published:** 2015-02-24

**Authors:** Caitlin Sedwick

**Affiliations:** Freelance Science Writer, San Diego, California, United States of America

## Abstract

The mechanism by which plants make starch—a vital foodstuff for billions of humans—is poorly understood, with a clear role for just one enzyme, granular binding starch synthase. A new study identifies a protein needed to recruit this enzyme to the starch granule. Read the Research Article.

During the daytime, plants capture the energetic proceeds of photosynthesis by building up a cache of starch granules. At night, plants can draw on their starch stores to fuel their metabolic needs. The most abundant component of starch, amylopectin, is a polymer composed of many short, highly branched chains of glucose molecules. The less abundant component of starch, amylose, has a simpler structure comprised of a single, unbranched chain of glucose molecules.

The structural characteristics of amylopectin and amylose lend them to very different uses. For instance, amylopectin is easily digested by humans and is therefore a major source of dietary glucose. It also has several industrial uses because its highly branched nature makes it an excellent thickener and texturant. By contrast, amylose can’t be digested well by people because its long glucose chains tend to repack together after cooking. Curiously, despite the obvious importance of starches, the mechanisms of starch synthesis are still not fully understood. In this issue of *PLOS Biology*, David Seung, Samuel Zeeman, and colleagues describe a protein with a newly identified role in the synthesis of amylose.

Scientists have identified four chain-elongating and two branching enzymes that work together to synthesize amylopectin chains. But so far, only one enzyme—granule bound starch synthase, or GBSS—has been found to synthesize amylose. Loss or inhibition of GBSS completely prevents synthesis of amylose. But Seung and colleagues wondered whether there might be other, as-yet-unidentified, proteins or genes important for starch production.

A promising candidate for such a gene was one first identified in the rockcress *Arabidopsis thaliana*, designated At5g39790, which encodes a protein that can bind to starches (Fig. 1). The protein also localizes to chloroplasts, where the plant stores its starch granules. Seung and colleagues set out to investigate its role in starch production and, on the basis of their findings, renamed At5g39790 to “Protein Targeting To Starch” (PTST).

**Fig 1:**
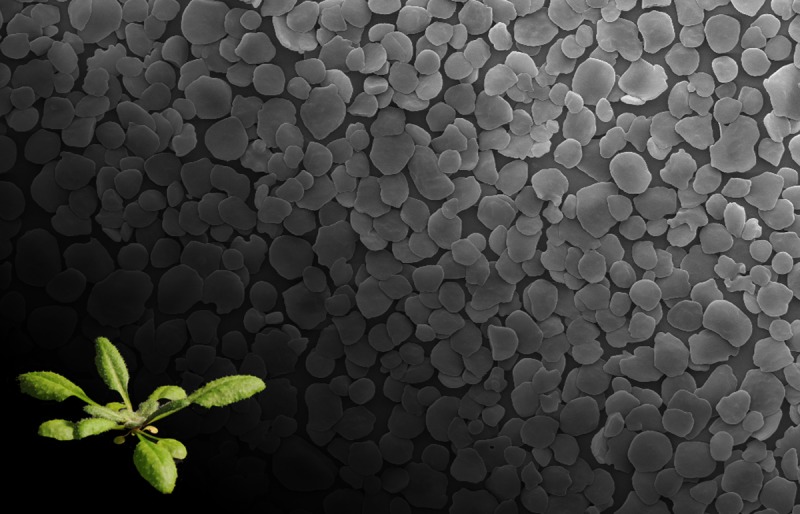
Starch granules are produced as a carbohydrate reserve in plants, including the model plant *A. thaliana*.

To determine whether At5g39790 might be involved in starch synthesis, Seung et al. studied plants carrying mutations that prevent the PTST protein from being made. Analysis of the starch content in mutant plants showed that although the total amount of starch is unchanged, the plants entirely lack amylose. This indicates that PTST is required for amylose production.

The only other gene currently known to be required for amylose synthesis is GBSS. GBSS is normally one of the most abundant proteins found in plant starch granules, but surprisingly, Seung and colleagues found that plants lacking PTST fail to localize GBSS to their starch granules. In fact, the PTST mutant plants have almost no GBSS protein at all.

The absence of GBSS explains why PTST-deficient plants do not make amylose, but why should loss of PTST affect GBSS protein levels? GBSS is thought to be highly unstable in the absence of starch, so the researchers sought to test whether PTST affects localization of GBSS to starch granules, where GBSS would be more stable. Observations of fluorescently tagged PTST and GBSS in live plants showed that GBSS requires PTST to be recruited to starch granules. Interestingly, Seung and colleagues observed that PTST also localizes to starch granules, albeit only transiently. PTST localization at starch granules is defective in the absence of GBSS, suggesting the two proteins rely on each other to reach granules.

Seeking an explanation for how these two proteins affect each other’s subcellular localization, the authors hypothsized that PTST may directly interact with GBSS. Consistent with this idea, analysis of sequence information for GBSS and PTST revealed that both proteins contain motifs called coiled-coils, which are known to mediate protein–protein interactions. Indeed, in vitro pull-down assays confirmed that PTST and GBSS bind to each other via their coiled-coil motifs, and immunoprecipitation experiments demonstrated that the two proteins could be pulled out together from intact plant cells. On the basis of these findings, Seung et al. concluded that the starch-binding ability of PTST allows this protein to localize to starch granules, and that thanks to its coiled-coil domain, PTST can bring GBSS along for the ride. Upon reaching the granule, PTST disassociates from both GBSS and starch, then relocalizes to the chloroplast stroma, while GBSS remains behind to synthesize amylose.

Taken together, these data reveal PTST to be important for amylose synthesis, expanding our knowledge of starch synthesis pathways in plants. This work may also prove important in other respects because until now, the only way to manipulate amylose levels in plants was to target GBSS directly. The identification of another component of the pathway promises another route to manipulating the relative amylose and amylopectin content of plants, with attendant implications for use of starches in both industrial and food science applications.
